# Transplantation of Mesenchymal Stem Cell-Derived Hepatocytes Primed with Quercetin Alone or in Combination with Rutin and LiCl Enhances Liver Regeneration

**DOI:** 10.3390/cells15131206

**Published:** 2026-07-02

**Authors:** Tuba Shakil Malick, Rida-E-Maria Qazi, Aisha Ishaque, Abiha Fatima, Irfan Khan, Shaheen Faizi, Asmat Salim, Farasat Zaman

**Affiliations:** 1 Stem Cell Research Laboratory, Dr. Panjwani Center for Molecular Medicine and Drug Research, International Center for Chemical and Biological Sciences, University of Karachi, Karachi 75270, Pakistan; tubashakil14@gmail.com (T.S.M.); ridaemaria@yahoo.com (R.-E.-M.Q.); aishaishaque123@yahoo.com (A.I.); abiha93@hotmail.com (A.F.); irfankhan.bangash@aku.edu (I.K.); 2 Centre for Regenerative Medicine and Stem Cell Research, The Aga Khan University Karachi, Karachi 74800, Pakistan; 3 College of Molecular Medicine, Ziauddin University, Karachi 75600, Pakistan; 4 HEJ Research Institute of Chemistry, International Center for Chemical and Biological Sciences, University of Karachi, Karachi 75270, Pakistan; shaheenfaizi@hotmail.com; 5 Department of Women’s and Children’s Health, Karolinska Institute, Biomedicum A4, 171 77 Stockholm, Sweden

**Keywords:** Wnt, MSCs, quercetin, rutin, lithium chloride, liver regeneration, hepatocytes

## Abstract

**Highlights:**

**What are the main findings?**

- Quercetin alone or in combination with rutin and lithium chloride (LiCl) promotes differentiation of mesenchymal stem cells (MSCs) into hepatocyte-like cells.
- Transplantation of quercetin-primed MSC-derived hepatocytes restores liver function and reduces inflammation and fibrosis in a rat bile duct ligation model.

**What are the implications of the main findings?**

- MSC-derived hepatocytes primed with quercetin alone or combined with rutin and LiCl exhibit enhanced liver regenerative potential.
- Ex vivo priming of MSC-derived hepatocytes with flavonoids and LiCl represents a new promising therapeutic strategy for liver regeneration.

**Abstract:**

Inhibition of Wnt/β-catenin signaling differentiates mesenchymal stem cells (MSCs) into hepatocytes. However, there is lack of data on whether transplanting these cells can enhance liver regeneration. Additionally, it remains unknown if the flavonoids quercetin and rutin and the clinically used drug lithium chloride (LiCl) can effectively differentiate MSCs into hepatocytes and promote liver regeneration. To address this, the rat bile duct ligation (BDL) fibrosis model was used. Male Wistar rats were surgically ligated. Liver function tests, histological analysis and cell tracking were also conducted to validate liver regeneration. Treatment with quercetin inhibited the Wnt pathway, while rutin and LiCl activated it. Hepatic differentiation was noted in three treatment groups: quercetin, quercetin with rutin and LiCl. We observed that the initial downregulation of the Wnt pathway, followed by its upregulation, facilitated the differentiation of MSCs into hepatocytes. Transplantation of quercetin-treated MSC-derived hepatocytes into the rat BDL fibrosis model resulted in complete restoration of liver function, normalization of elevated systemic liver enzymes and reduction of inflammation and fibrosis. Interestingly, quercetin-treated hepatocytes resulted in enhanced liver regeneration compared to rutin and LiCl. Finally, tracking of labeled hepatocytes confirmed their main localization in the liver. In conclusion, MSC-derived hepatocytes, generated through ex vivo treatment with quercetin, exhibit enhanced liver regeneration. These findings provide a novel ex vivo treatment strategy with flavonoids and the clinically used drug LiCl to achieve enhanced liver regeneration in vivo.

## 1. Introduction

End-stage liver diseases are major health issues globally, with nearly 2 million people dying each year due to chronic liver diseases [1]. The liver, being the largest organ in the body, is responsible for various critical functions [2]. Any damage to the liver can result in serious, life-threatening complications. Despite numerous advancements in modern medicine, there is currently no effective treatment available for liver cirrhosis, apart from liver transplantation. Stem cell-based therapy has generated considerable interest in the scientific community as a potential alternative to liver transplantation [3]. Stem cells can self-renew and differentiate into any cell type, facilitating growth, healing, and tissue replacement. Among various types of stem cells, mesenchymal stem cells (MSCs) are commonly used in regenerative medicine due to their multilineage differentiation potential.

Various approaches, such as the use of growth factors, cytokines and small molecules, have been employed to enhance MSCs’ differentiation toward hepatocytes [4]. Small-molecule-based approaches offer a significant advantage over growth factors. In this context, signaling pathway modulators provide an additional advantage by targeting specific signaling molecules. Several studies have emphasized the role of signaling pathways in guiding stem cells toward specific cell lineage commitments. The Wnt/β-catenin pathway, in particular, plays a crucial role in stem cell differentiation into specific lineages by regulating proliferation, differentiation, and apoptosis [5,6,7]. Precise regulation of the Wnt pathway is essential to avoid potential pathological complications like rheumatoid arthritis, osteoporosis, and cancer [8].

Lithium chloride, a known Wnt signaling activator, inhibits GSK-3 and activates Wnt signaling [9], while Quercetin interacts with β-catenin and inhibits the binding of β-catenin to TCF, leading to the inactivation of the Wnt/β-catenin signaling pathway [10]. Although rutin-induced Wnt pathway modulation is not well known, it has been reported that troxerutin, a synthetic precursor of rutin, activates the Wnt pathway [11].

In cases of irreversible end-stage liver disease, chemotherapy and liver transplantation are the only treatment options. Despite impressive advancements in the development of new drugs and stem cell transplantation, no treatment currently exists that can improve liver function or promote liver cell proliferation. Flavonoids possess a variety of biological activities, including antioxidant [12], antiviral [13], antibacterial [14], and anti-inflammatory [15]. Given the critical role that Wnt signaling plays in the differentiation process, it can be deemed an important therapeutic target [16,17,18]. Therefore, small molecules and bioactive natural compounds capable of modulating Wnt signaling can be harnessed to design new therapeutic strategies [19].

The current study aimed to investigate whether modulation of the Wnt pathway using the flavonoids quercetin and rutin and a clinically used drug LiCl can drive MSCs’ differentiation into hepatocytes and foster liver regeneration in a rat bile duct ligation (BDL) fibrosis model. Our study presents a novel strategy for differentiating MSCs into hepatocytes, which led to enhanced liver regeneration in rats. This approach is safer and more efficient, providing a quicker method to guide MSC differentiation towards hepatocytes for potential use in liver regeneration therapies.

## 2. Experimental Work

### 2.1. Chemical Compounds

Rutin and quercetin were provided by Prof. Dr. Shaheen Faizi at the HEJ Research Institute of Chemistry, International Center for Chemical and Biological Sciences, University of Karachi. LiCl was purchased from Sigma-Aldrich, Burlington, MA, USA (cat no# 7447-41-8). Quercetin and rutin were resuspended in DMSO while LiCl was resuspended in sterile water.

### 2.2. Animals

Experimental animals were used after ethical approval from the local institutional ethical committee, Institutional Animal Care and Use Committee, Dr. Panjwani Center for Molecular Medicine and Drug Research, International Center for Chemical and Biological Sciences, University of Karachi (ASP # 2020-005; 11 January 2020). Experimental procedures were carried out according to the international guidelines for care and use of laboratory animals. Wistar rats of both sexes weighing 160–180 g (2–3 months) were used to isolate bone marrow MSCs, while healthy Wistar male rats weighing 250–280 g (5–6 months) were used to develop the BDL fibrosis model. Rats that showed any sign of infection were excluded from the study. All rats were kept in individual cages in a room maintained under a 12 h light:12 h dark cycle, 22 ± 2 °C temperature and 55 ± 5% relative humidity and provided with free access to a standard chow diet and water.

### 2.3. Isolation and Passaging of Bone Marrow-Derived MSCs

MSCs were isolated from rat bone marrow by flushing the bone marrow with complete medium, DMEM, containing 10% fetal bovine serum, 100 units/mL penicillin, 100 μg/mL streptomycin, and 1 mmol/L sodium pyruvate. The cells were cultured under standard conditions in a CO_2_ incubator at 37 °C for 5 days. The floating cells were removed, and the MSCs were sub-cultured once they reached 70–80% confluence to obtain a homogenous population of MSCs.

### 2.4. Characterization of MSCs

To confirm the isolation of MSCs, immunocytochemistry and flow cytometry were performed to detect cell surface markers including CD 29, CD44, vimentin, CD90, CD117, and CD45. Trilineage differentiation ability of MSCs was evaluated by culturing the cells separately in adipogenic induction medium, osteogenic induction medium, and chondrogenic induction medium. After 21 days, the cells were washed and stained with Oil Red O, Alizarin Red S, and Alcian Blue to analyze adipogenesis, osteogenesis, and chondrogenesis, respectively. All these experiments were performed according to the protocol described previously [20]. The stained cells were observed under a phase contrast microscope (TE 2000E, Nikon, Tokyo, Japan).

### 2.5. Cytotoxicity Assay

Different concentrations of quercetin, rutin, or LiCl were subjected to JC-1 assay to determine the non-cytotoxic concentration of these compounds. The percentage of apoptotic cells was analyzed using BD Cell Quest Pro software (version 5.1).

### 2.6. Analysis of Wnt Pathway

To analyze the Wnt pathway modulation after treatment with quercetin, rutin, or LiCl, cells were treated with these compounds for 24 h. RNA was isolated using Trizol reagent according to the manufacturer’s instructions. RNA quantification was done at 260 nm through a UV spectrophotometer spectrophotometer (NanoDrop 2000, Thermo Scientific, Waltham, MA, USA), followed by cDNA synthesis through a RevertAid First Strand cDNA Synthesis Kit (Thermo Scientific, Waltham, MA, USA), as per the manufacturer’s instructions. The Mastercycler CFX96 Touch Real-Time PCR Detection System (Bio-Rad, Hercules, CA, USA) was used for amplification. CFX maestro software (Version 2.3) was used to set a program in which 95 °C temperature was provided for 10 min for initial denaturation followed by 40 cycles of denaturation at 95 °C for 30 s, and annealing at 58 °C for 1 min, to amplify the cDNA. Glyceraldehyde-3-phosphate dehydrogenase (GAPDH) was used as the housekeeping gene and fold change (2^−ΔΔCt^) was calculated. A list of all primers used in this study is mentioned in Table 1. The Wnt pathway was also analyzed by immunocytochemistry using β-catenin antibody (Thermo Scientific, Waltham, MA, USA). Fluorescence was analyzed through a fluorescence microscope.

### 2.7. In Vitro Induction of Hepatic Differentiation

MSCs were treated with 10 µM quercetin, 10 µM rutin, or 10 mM LiCl for 24 h in serum-free medium. Then medium containing compound was removed, and complete medium (without test compound) was added and incubated for an additional 14 days. To study combination effects, MSCs were first treated with one compound in serum-free medium for 24 h, and then medium containing the compound was removed and complete medium was added and incubated for 10 days, followed by treatment with the second compound for 24 h in serum-free medium. Then, medium containing the compound was removed and complete medium was added and incubated for the next 4 days. So, in all cases, there was a total of 14 days of incubation Table 2.

### 2.8. Analysis of Hepatic Differentiation

For the analysis of hepatic differentiation of MSCs at gene level, RNA was isolated from the treated and untreated cells after the completion of specified incubation periods. cDNA was synthesized and hepatic differentiation was analyzed using hepatic gene primers (Table 1). Periodic acid Schiff and Oil Red O staining were performed according to the manufacturer’s instructions. Cells were observed under a bright-field microscope (TE-2000E, Nikon, Japan). To validate gene expression and staining results, immunocytochemical analysis was performed in three treatment groups—quercetin, quercetin + rutin, and quercetin + LiCl—and in control MSCs. Primary antibodies specific to alpha fetoprotein (AFP), albumin, hepatocyte nuclear factors (HNF), cytokeratin 18 (CK-18), and tyrosine aminotransferase (TAT) were used at a dilution ratio of 1:50 to verify hepatic differentiation.

### 2.9. Bile Duct Ligation (BDL) Model for Liver Fibrosis

Wistar rats (250–300 g) were used to develop liver fibrosis models. All rats were maintained under standard conditions with free access to a standard chow diet and water.

A single animal was considered as an experimental unit. After a few days of acclimatization in the animal house research facility of ICCBS, rats were randomly divided into groups to avoid any potential confounding factors. Three animals per group were allocated (*n* = 3) for a total of six study groups (total eighteen animals): sham control, BDL fibrosis model, BDL fibrosis models transplanted with MSCs, quercetin, quercetin + rutin, or quercetin + LiCl-treated MSCs. Surgical methods were chosen to induce liver fibrosis in rats because it is a highly reproducible and precise method. To induce the BDL fibrosis model, rats were anesthetized, and horizontal incisions were made to open the abdomen. The liver was exposed and elevated, followed by placement of a single knot of chromic catgut suture (4-0) around the common bile duct for ligation. Saline solution was used to rinse the peritoneal cavity, and both abdominal layers were closed with 5-0 proline sutures. Subcutaneous injections of diclofenac sodium and antibiotics were administered. Post-procedure, ocular and muscular reflexes were monitored until the rats fully regained consciousness. Daily observations were conducted to assess mobility and detect any signs of discomfort. On the 10th day after surgery, MSCs were transplanted via the tail vein. The cells were trypsinized and washed three times with 1× PBS, and one million cells were resuspended in 200 μL of 1× PBS for transplantation. The rats were sacrificed 10 days after transplantation.

Blood samples were collected for biochemical analysis, while the liver was harvested for histological analysis. Rats without ligation served as sham control. Rats with hemorrhage or accidental transection in the bile duct which led to uncontrolled leakage were excluded from the study.

The METAVIR scoring system was used to assess the extent of inflammation and fibrosis.

### 2.10. Blood Profile and Histological Analysis

Liver function was assessed in all experimental groups by analyzing blood profile, including direct bilirubin, indirect bilirubin, total bilirubin, gamma glutamyl transferase (GGT), and serum glutamate pyruvate transaminase (SGPT), using an automated analyzer. The liver was harvested, processed, and sectioned by following the standard protocol. The sections were then stained with H&E and Masson’s trichrome stains, by following the manufacturer’s instructions. Stained sections were subsequently analyzed for fibrosis and inflammation and scored according to the Metavir scoring system. Finally, the stained liver sections were examined under a bright-field microscope. Percentage of fibrotic area was measured using Fiji imageJ software version 1.54 (National Institutes of Health, Bethesda, MD, USA).

### 2.11. Tracking of Transplanted Cells

Treated and untreated MSCs were labeled with the DiI dye according to the manufacturer’s instructions and transplanted through the tail vein in the BDL model. Ten days after the transplantation, rats were perfused as described previously [20]. Different tissues, i.e., liver, kidney, heart, and spleen, were harvested and fixed in 4% PFA for 4 h. Tissue blocks were prepared through OCT cryosectioning medium and 10 µm tissue sections were made using cryotome (Thermo Scientific, Waltham, MA, USA).

### 2.12. Statistical Analysis

SPSS software version 20.0 was used to perform statistical analysis to determine the results’ significance. One-way ANOVA with Bonferroni post hoc test was used to compare multiple groups, while an independent sample *t*-test was used to compare two groups. A *p*-value of less than 0.05 was considered statistically significant.

## 3. Results

### 3.1. Isolation and Differentiation of MSCs

**Figure 1:**
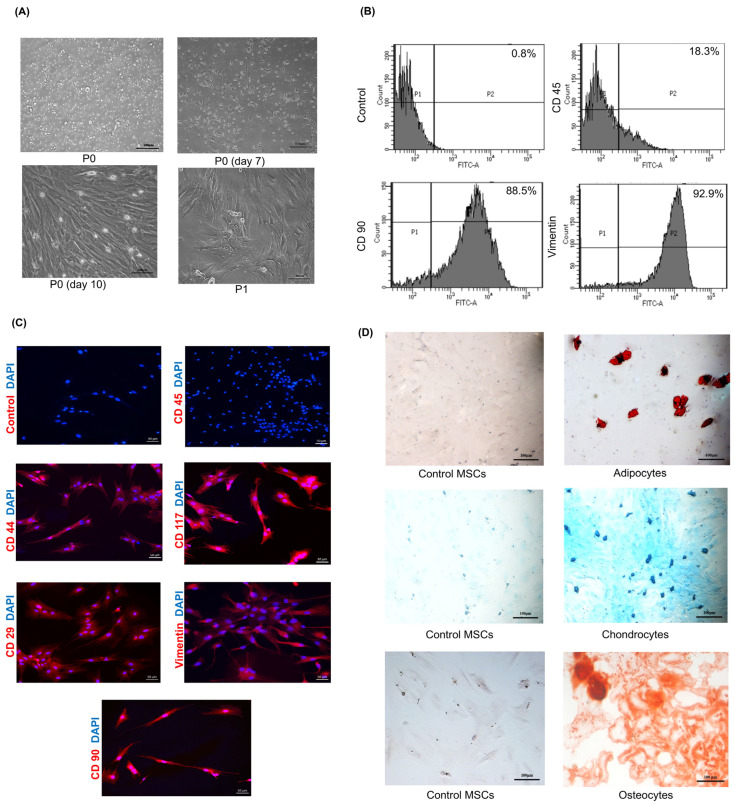
Isolation and characterization of MSCs: (**A**) Rat bone marrow culture indicating the heterogeneous population of cells on the day of isolation at passage 0 (P0), MSCs exhibiting fibroblast-like morphology on day 7 and day 10, and sub-cultured to get the homogenous population at passage 1 (P1). (**B**) Immunocytochemistry showing positive expression (red fluorescence) of CD29, CD44, CD117, vimentin, and CD90 and negative expression of CD45 (a hematopoietic marker). Blue fluorescence indicates DAPI-stained nuclei. (**C**) Flow cytometric analysis showing positive expression of CD90 and vimentin and negative expression of CD45. (**D**) Trilineage differentiation of MSCs into adipocytes, chondrocytes and osteocytes. Trilineage differentiation potential was evidenced by the presence of oil droplets as indicated by Oil Red O staining, suggesting adipogenic differentiation. Alcian blue-stained cells demonstrated glycosaminoglycans, confirming chondrogenic differentiation, while Alizarin Red S staining revealed calcium deposits, indicating osteogenic differentiation in the induced MSCs.

We first validated the differentiation potential of isolated MSCs. These cells exhibited spindle-shaped and fibroblast-like morphology as anticipated (Figure 1A). Immunocytochemical analysis revealed positive expression of mesenchymal markers CD29, CD44, vimentin, CD90, and CD117 alongside negative expression of the hematopoietic marker CD45 (Figure 1B). Similarly, flow cytometric analysis showed CD90 and vimentin-positive cells with negative expression of CD45, as compared with the isotype control (Figure 1C). MSCs were also successfully differentiated towards adipocytes, chondrocytes and osteocytes (Figure 1D).

### 3.2. Cytotoxicity Analysis of Quercetin, Rutin and Lithium Chloride

**Figure 2:**
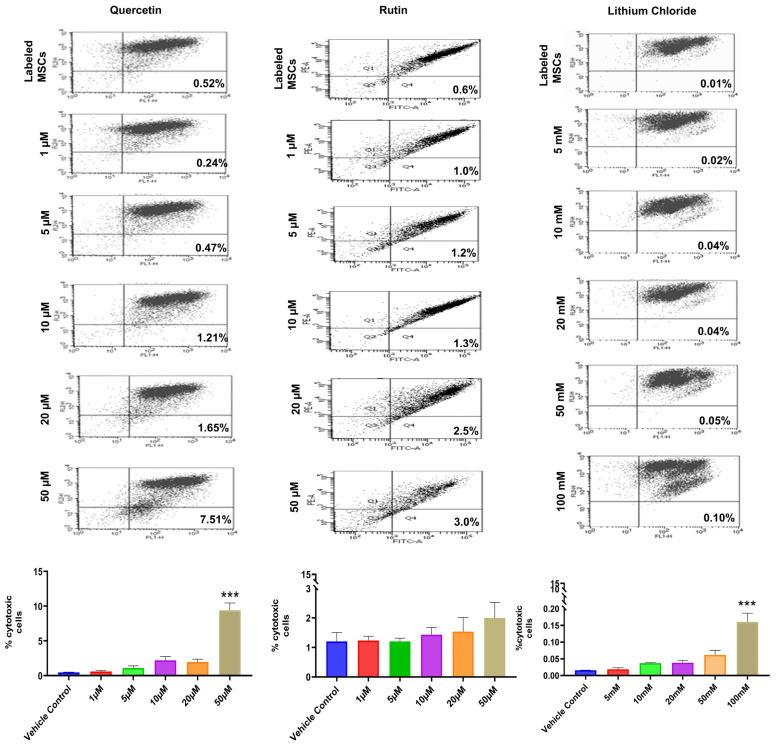
Cytotoxicity analysis of MSCs: Dot plots of JC1 flow cytometry analysis showing the majority of unstained cells in lower left quadrant and mixed cell population in upper right quadrant; the lower right quadrants represent the apoptotic cells after treatment of MSCs with quercetin, rutin, or lithium chloride (LiCl). Bar graphs representing the dose-dependent increase in cell cytotoxicity with significant increase in 50 μM quercetin- and 100 mM LiCl-treated MSCs, while no significant cytotoxicity was observed in rutin-treated MSCs at all concentrations. Data is represented as mean ± S.E.M., where *n* = 3. Statistical significance is indicated by *p* value: *** = *p* < 0.001.

Next, cellular toxicity was measured in vitro. A dose-dependent increase in cytotoxicity was observed, with 50 µM quercetin and 100 mM LiCl (*p*-value < 0.01), while no significant cytotoxicity was noted in rutin-treated MSCs (Figure 2). Based on these cytotoxic data, safe, non-cytotoxic concentrations of 10 µM quercetin, 10 µM rutin, and 10 mM LiCl were selected for subsequent experiments.

### 3.3. Modulation of Wnt Pathway by Quercetin, Rutin and LiCl

**Figure 3:**
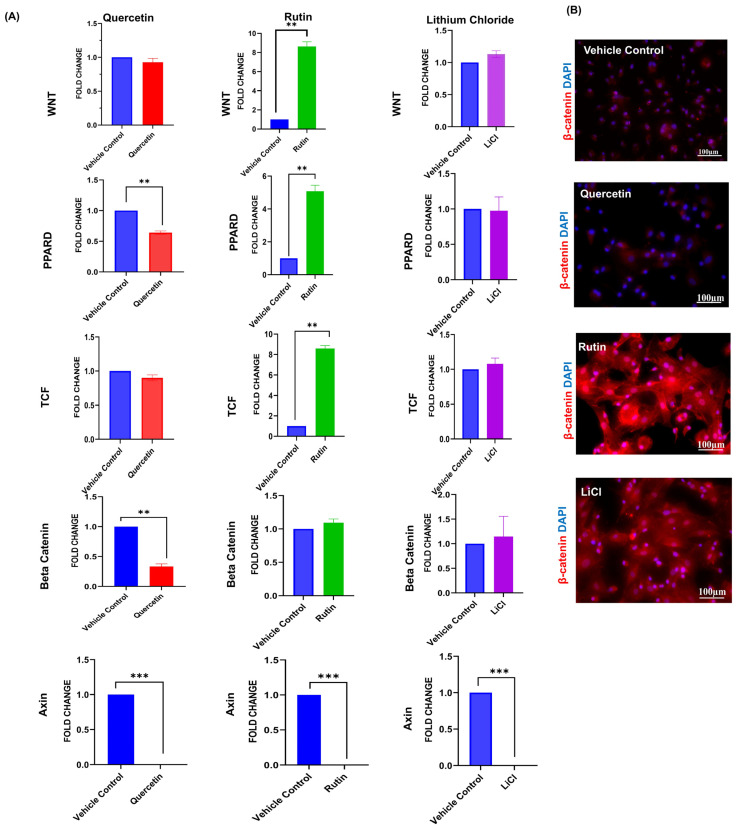
Wnt pathway analysis: (**A**) Bar graphs depicting the quantitative fold change (2^−ΔΔCT^) expression of various Wnt pathway genes after 24 h treatment with quercetin, rutin or LiCl determined by qPCR analysis. (**B**) Immunocytochemistry showing the expression of β-catenin (stained in red) in all treated and untreated groups. Blue fluorescence indicates DAPI-stained nuclei. Data is represented as mean ± S.E.M., where *n* = 3. Statistical significance is indicated by *p* values: ** = *p* < 0.01 and *** = *p* < 0.001.

To investigate Wnt pathway modulation, corresponding genes were examined using qPCR. Compared with control MSCs (vehicle), quercetin-treated cells showed downregulation of *peroxisome proliferator-activated receptor (PPARD)* (*p*-value < 0.01), *β-catenin* (*p*-value < 0.01), and *axin* (*p*-value < 0.001), suggesting suppression of the Wnt pathway (Figure 3A). However, rutin-treated MSCs displayed downregulation of *axin* (*p*-value < 0.001) and upregulation of *PPARD* (*p*-value < 0.01) and *TCF* (*p*-value < 0.01).

Similarly, LiCl-treated MSCs exhibited downregulation of *axin* (*p*-value < 0.001) but no significant change in *PPARD*, *TCF*, *Wnt*, and *β-catenin* (Figure 3A). In summary, these results indicated that quercetin downregulated the Wnt pathway, while rutin and LiCl activated it. Immunocytochemistry analysis also demonstrated intense β-catenin staining in rutin- and LiCl-treated cells but not in quercetin-treated cells, as compared to control MSCs (Figure 3B).

### 3.4. Quercetin Alone and in Combination with Rutin or LiCl Differentiates MSCs into Hepatocytes

**Figure 4:**
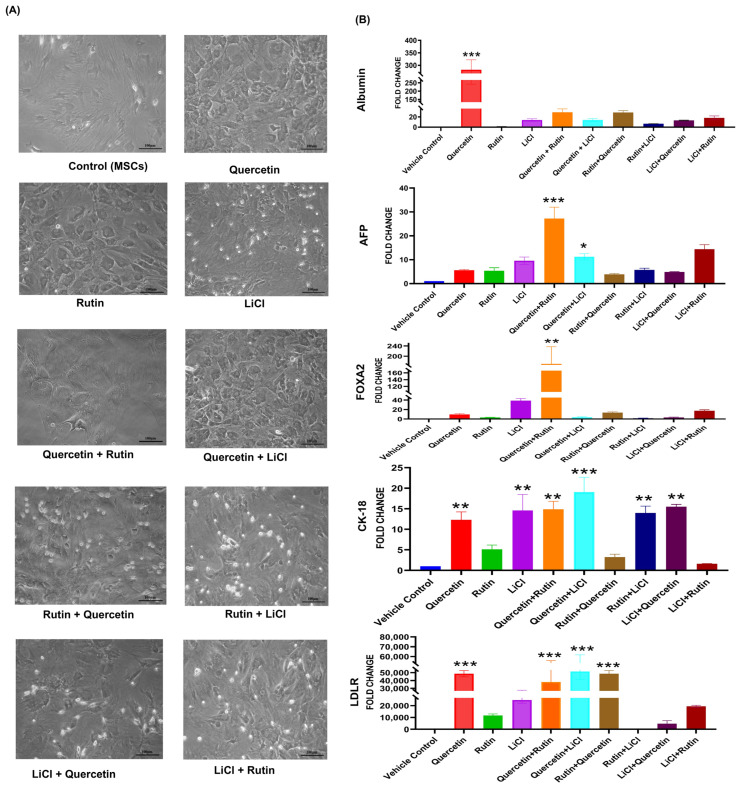
Analysis of hepatic differentiation of MSCs: (**A**) Morphological analysis of treated MSCs showing polygonal shape morphology with diploid nuclei in quercetin-, quercetin + rutin-, and quercetin + LiCl-treated cells, while the rest of the groups did not show hepatic morphology. Images were acquired with a phase contrast microscope. (**B**) Bar graphs representing quantitative fold change (2^−ΔΔCT^) expression of hepatic markers determined by qPCR analysis. Data is represented as mean ± S.E.M., where *n* = 3. Statistical significance is indicated by *p* values: *** = *p* < 0.001, ** = *p* < 0.01 and * = *p* < 0.05.

On day 14 of culture, cells treated with quercetin, quercetin + rutin and quercetin + LiCl exhibited hepatocyte-like morphology characterized by polygonal shape and diploid nuclei (Figure 4A). Gene expression analysis revealed the expression of *albumin*, *Alpha-fetoprotein (AFP)*, *Forkhead box protein A2 (FoxA2)*, *CK-18* and *low-density lipoprotein receptor (LDLR)* in all treated groups (Figure 4B).

The highest expression of *albumin* (*p*-value < 0.001) was observed in quercetin-treated cells. *AFP* was upregulated in all the treated cells, with a significant increase seen in quercetin + rutin- (*p*-value < 0.001) and quercetin + LiCl-treated cells (*p*-value < 0.05). All treated cells exhibited increased *FoxA2* expression, with a significant increase noted in the quercetin + rutin (*p*-value < 0.001) treatment group. A significantly higher *CK-18* expression was observed in all treated cells except rutin-, rutin + quercetin-, and LiCl + rutin-treated cells. Additionally, LDLR was significantly upregulated in quercetin-, quercetin + rutin-, quercetin + LiCl-, and rutin + quercetin-treated cells compared to untreated cells (*p*-value < 0.001) (Figure 4B).

**Figure 5:**
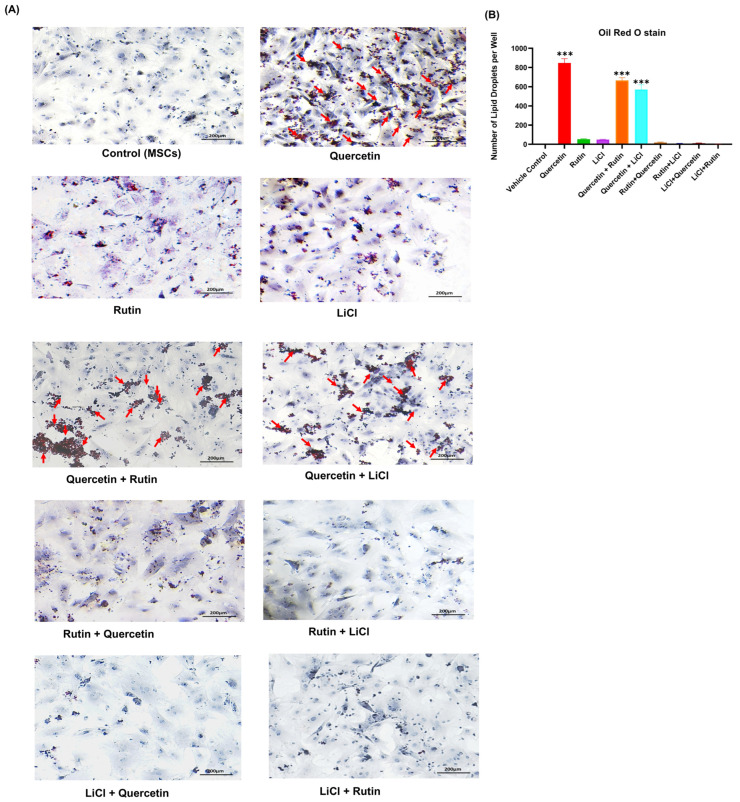
Oil Red O staining of treated cells: Images showing (**A**) numerous oil droplets (indicated by red arrows) in quercetin-, quercetin + rutin-, and quercetin + LiCl-treated cells. (**B**) Quantitative analysis of oil droplets in treated cells performed by ImageJ software, version 1.54g (National Institutes of Health, USA) Data is represented as mean ± S.E.M., where *n* = 3. Statistical significance is indicated by *p* value: *** = *p* < 0.001.

**Figure 6:**
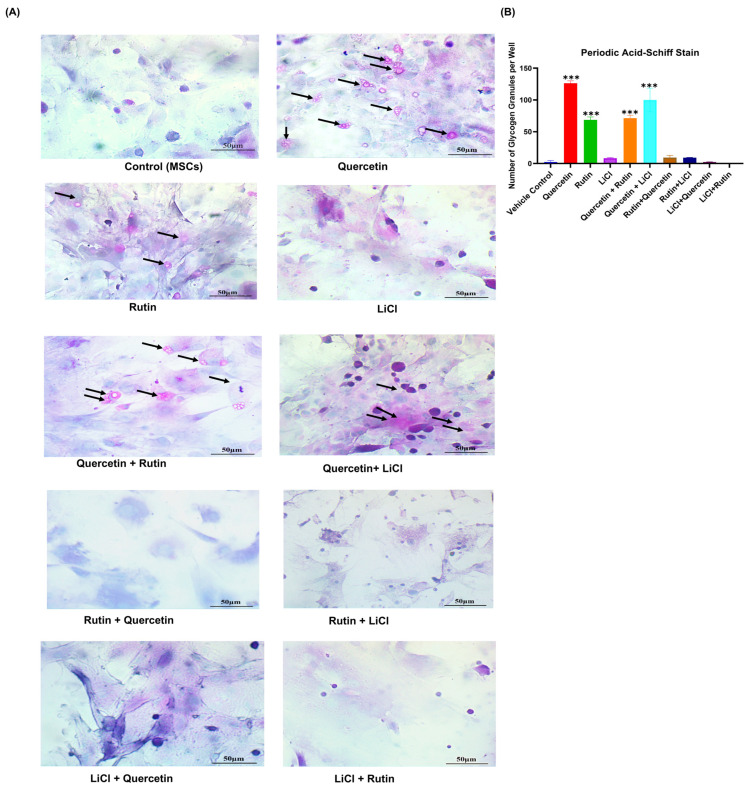
Periodic acid Schiff staining: Images showing (**A**) numerous glycogen granules (indicated by black arrow). (**B**) Bar graph representing the quantitative analysis of glycogen granules in all groups performed by ImageJ software. Data is represented as mean ± S.E.M., where *n* = 3. Statistical significance is indicated by *p* value: *** = *p* < 0.001.

**Figure 7:**
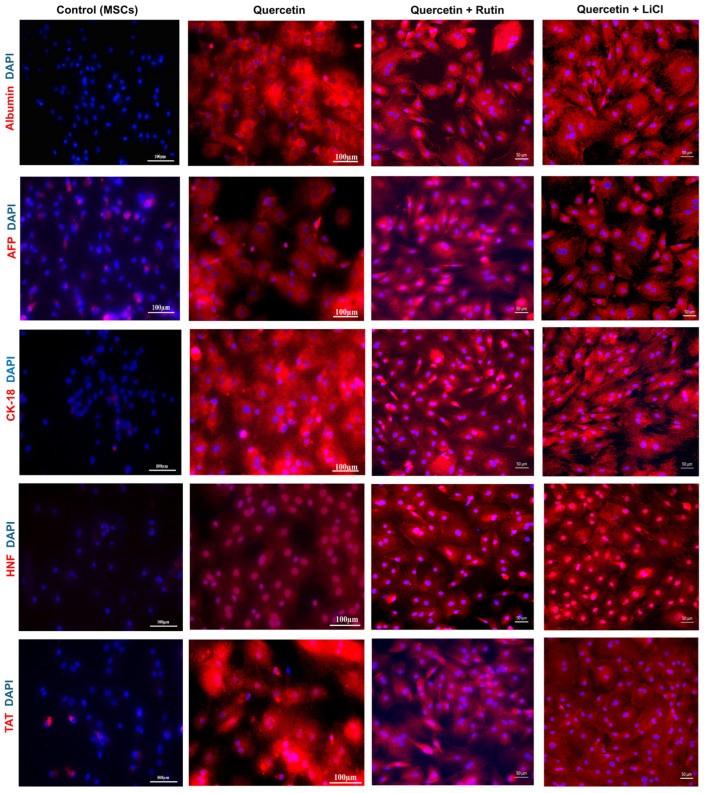
Immunocytochemistry of MSC-derived hepatocytes: Fluorescence imaging (red staining) showing the positive expression of albumin, alpha fetoprotein (AFP), cytokeratin-18 (CK-18), hepatocyte nuclear factor (HNF), and tyrosine aminotransferase (TAT) in treated MSCs (MSC-derived hepatocytes), compared with untreated (control) MSCs. Blue fluorescence indicates DAPI-stained nuclei.

Numerous oil droplets were observed in quercetin-, quercetin + rutin-, and quercetin + LiCl-treated cells stained with Oil Red O stain (*p*-value < 0.001) (Figure 5A,B). Similarly, glycogen granules were observed in quercetin-, quercetin + rutin-, quercetin + LiCl-, and rutin-treated cells (*p*-value < 0.001) (Figure 6A,B). In summary, these results revealed that treatment of MSCs with quercetin and the combination of quercetin with rutin and LiCl stimulated more hepatic differentiation than other groups. Finally, the immunohistochemistry for albumin, AFP, CK-18, HNF and TAT also confirmed the hepatic differentiation of the treated cells (Figure 7).

### 3.5. Transplantation of MSC-Derived Hepatocytes Treated with Quercetin, Rutin and LiCl Prevents Inflammation and Fibrosis In Vivo

**Figure 8:**
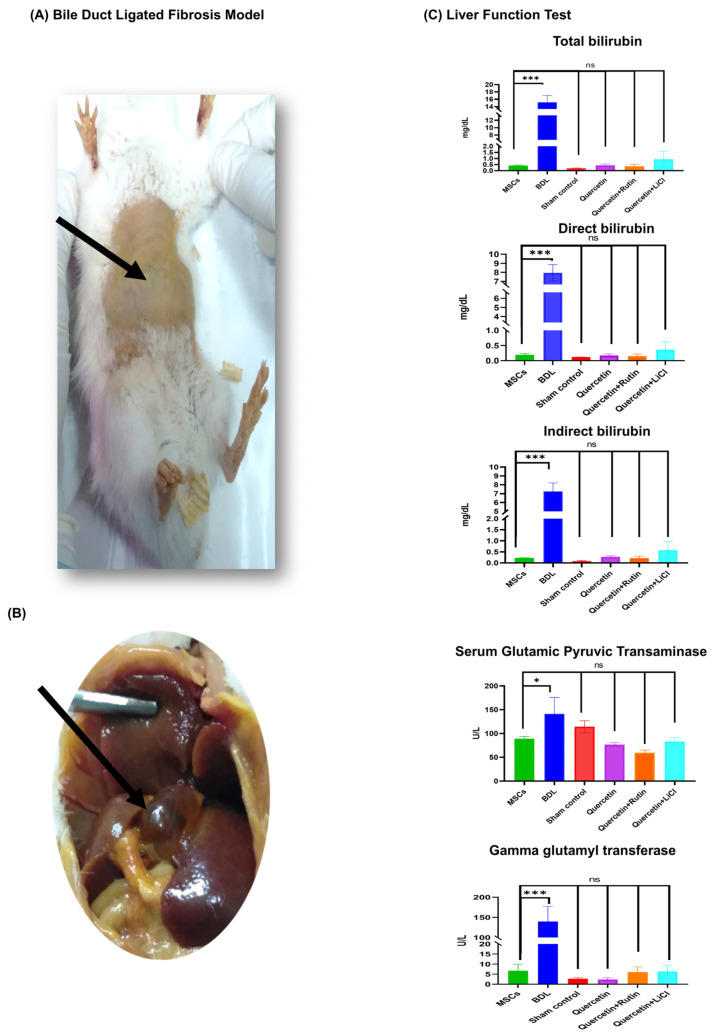
Gross appearance of in vivo disease model and biochemical analysis of liver function: Bile duct ligation (BDL) in rat models showing (**A**) pale body, and (**B**) yellow coloration of liver and inflamed bile duct (black arrow). (**C**) Bar graphs represent the comparison of liver enzymes among all animal groups. Data is represented as mean ± S.E.M.; *n* = 3. Statistical significance is indicated by *p* values: *** = *p* < 0.001 and * = *p* < 0.05, not significant (ns).

We next performed the transplantation of MSC-derived hepatocytes in vivo. The rat BDL fibrosis model was used, which exhibited signs of fibrosis on the 10th day following surgery, as anticipated. The body turned a pale hue, and the liver appeared yellow, while the bile duct swelled like a balloon due to bile accumulation (Figure 8A,B).

To verify liver regeneration, we measured circulating liver enzyme levels. Hepatic function tests showed a significant increase in these enzymes in BDL animals, consistent with liver injury. In contrast, enzyme levels remained stable across all treated groups, showing no comparable increase relative to the mesenchymal stem cell (MSC) group (Figure 8C).

Masson’s trichrome staining was used to analyze fibrosis, while H&E staining was employed to identify structural alteration and inflammation. The METAVIR scoring system, which assesses the degree of inflammation (A0—no activity; A1—mild activity; A2—moderate activity; A3—severe activity) and extent of fibrosis (F0—no fibrosis; F1—portal fibrosis; F2—periportal fibrosis; F3—bridging fibrosis; F4—cirrhosis), was employed.

**Figure 9:**
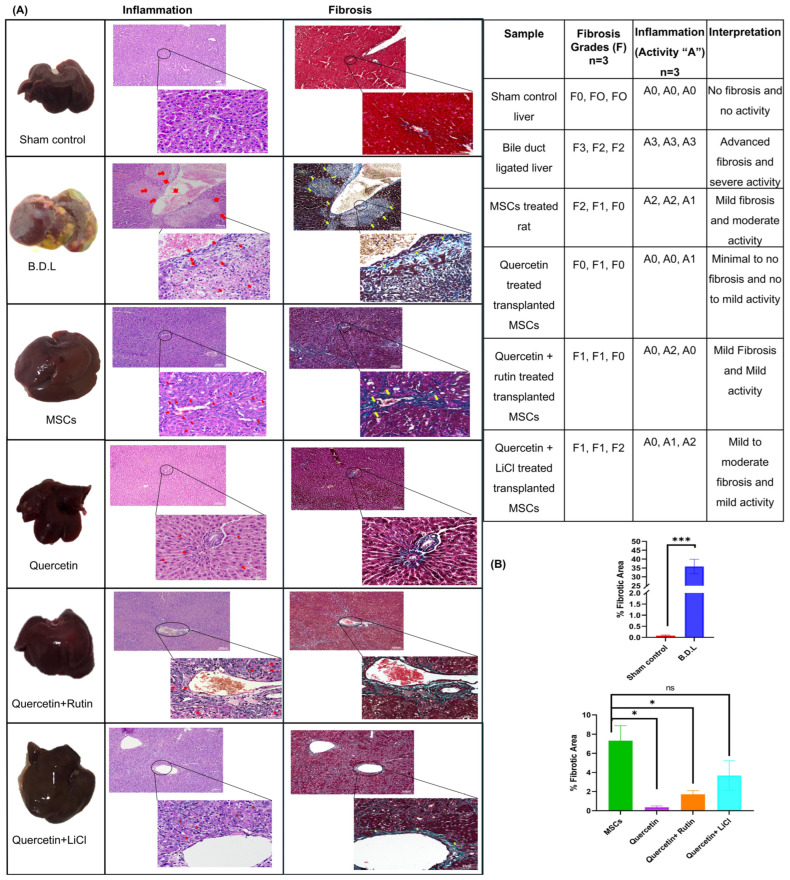
Histological analysis: (**A**) Histological analysis of liver showing inflammation (indicated by red arrows) in H&E-stained sections and fibrosis (indicated by yellow arrows) in Masson’s trichrome-stained sections. The attached table of the Metavir scoring system demonstrates the degree of inflammation and fibrosis of livers in all animal groups used in the study. (**B**) Bar graphs representing the percentage of fibrotic area. Data is represented as mean ± S.E.M.; *n* = 3. Statistical significance is indicated by *p* value: *** *p* < 0.001, * = *p* < 0.05 and not significant (ns).

Histological analysis (Figure 9A) revealed that the sham control group exhibited normal hepatic morphology with no inflammation or no fibrosis. However, in the BDL group, there was a loss of hepatocytes and a significant infiltration of inflammatory cells around the portal track, resulting in severe inflammation, and advanced fibrosis score. In comparison, animals transplanted with MSCs (vehicle) demonstrated hepatic regeneration, reduced fibrosis, and less inflammation.

The transplantation of quercetin-treated MSC-derived hepatocytes resulted in enhanced hepatic regeneration with reduced inflammation and no fibrosis, compared to the MSC group (vehicle). Animals transplanted with quercetin + rutin- and quercetin + LiCl-treated MSC-derived hepatocytes also exhibited better regeneration than MSCs (vehicle) (Figure 9A). The fibrotic area was also reduced in all treated groups as compared to MSCs (*p*-value < 0.05) (Figure 9B).

### 3.6. Tracking of MSC-Derived Hepatocytes After Transplantation

**Figure 10:**
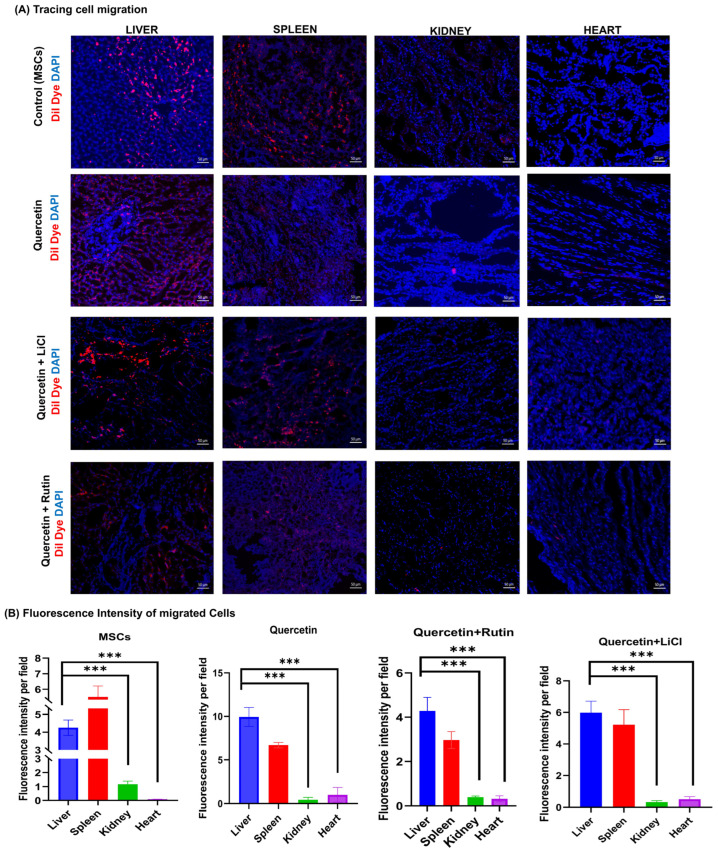
Tracking of transplanted cells: (**A**) Fluorescence images, showing the DiI-labeled cells (red) in liver, spleen, kidney, and heart, indicating migration of transplanted cells. Blue fluorescence indicates DAPI-stained nuclei. (**B**) Bar graphs representing the fluorescence intensity of transplanted cells per field. Data is represented as mean ± S.E.M., where *n* = 3. Statistical significance is indicated by *p* value: *** = *p* < 0.001.

Cellular tracking unveiled that the majority of labeled cells were in the liver and spleen, with only a sparse presence in the kidney and heart. These findings imply that both treated and untreated transplanted MSCs effectively migrated towards the injury site, actively participating in liver regeneration (Figure 10A,B). Notably, cells treated with quercetin exhibited explicit localization within the liver and displayed more intense staining than all other groups.

## 4. Discussion

We here provide the first-ever evidence that MSC-derived hepatocytes, obtained through Wnt inhibition, effectively regenerate the liver, prevent inflammation and fibrosis and normalize disturbed liver enzymes in a rat fibrosis model of bile duct ligation. The flavonoids quercetin and rutin, along with the clinically used drug LiCl, successfully differentiated MSCs into hepatocytes. Ex vivo treatment of MSCs with quercetin, either alone or in combination with rutin or LiCl, resulted in enhanced liver regeneration compared to the sham group or untreated MSCs. Notably, treatment with quercetin showed the most significant liver regeneration among all groups.

The Wnt signaling pathway plays a vital role in hepatic fate determination [21]. Previously, small molecules have been used alone or in combination with growth factors to demonstrate the significance of the Wnt pathway in hepatic differentiation. For instance, activation of Wnt signaling has been reported to induce hepatic differentiation and liver regeneration [22,23]. However, no data is available on whether hepatocytes obtained through Wnt inhibition can promote liver regeneration post-transplantation. Addressing this gap in knowledge, we isolated rat bone marrow MSCs treated with quercetin alone and in combination with rutin or LiCl. Prior to treatment, we determined the optimal concentration of each compound through cytotoxicity analysis.

Our findings revealed that quercetin inhibited the Wnt pathway, while rutin and LiCl activated it, corroborating prior reports that LiCl enhances cell proliferation and upregulates the Wnt pathway [24]. In contrast, quercetin has been reported to inhibit the Wnt pathway and decrease the proliferation of MSCs derived from the umbilical cord [25]. LiCl has previously been reported to activate the Wnt pathway in neuronal stem cells [24] and rescue bones from growth retardation [26]. Interestingly, it protects cholestatic hepatocytes from bile acid-mediated damage by blocking the NMDAR-GluNN2B subunit [27]. Quercetin has also been shown to downregulate Wnt signaling in cancer cells [28]. Seo et al. reported that rutin can enhance cellular movement of HaCaT keratinocytes through Wnt/β-catenin signaling [29]. Similarly, rutin is also known to promote antioxidative stress ability, proliferation and osteogenic differentiation [30]. These contrasting effects underscore the diverse impact of these compounds on different cell types and highlight their potential therapeutic relevance in distinct biological contexts.

Subsequent analysis explored whether treatment with quercetin, rutin and LiCl alone or in combination could induce hepatic differentiation in MSCs. We found that quercetin treatment significantly upregulated albumin, a core protein produced by hepatocytes [31]. AFP was notably elevated in cells treated with quercetin + rutin and quercetin + LiCl. AFP is a key glycoprotein expressed during fetal liver development. FoxA2, an essential transcription factor during liver development [32], was significantly upregulated by quercetin alone or in combination with rutin. Similarly, CK-18 and LDLR were also significantly upregulated in cells treated with quercetin, quercetin + rutin and quercetin + LiCl. CK-18 is a hepatic marker during the mid–late and late stages of hepatocyte differentiation [33], while LDLR is crucial for cholesterol transport. Altogether, these key biomarkers of hepatic differentiation were markedly upregulated following treatment with quercetin alone or in combination with rutin or LiCl, as evidenced by biochemical staining showing the presence of oil droplets and glycogen granules indicative of hepatic differentiation. Immunocytochemistry analysis further confirmed the expression of hepatic markers (albumin, AFP, CK-18, HNF, and TAT) in groups treated with quercetin, quercetin + rutin, and quercetin + LiCl.

Building on positive in vitro results, cells pre-treated with quercetin alone or in combination with rutin or LiCl were used for in vivo transplantation in a rat BDL fibrosis model. Bile duct ligation was surgically performed to induce liver fibrosis, and after ten days, rats exhibited symptoms of liver fibrosis. The rats’ bodies turned pale in color and the bile duct was inflamed. The yellow color of the liver was prominent during harvesting. The transplantation of MSC-derived hepatocytes treated with quercetin alone or combined with LiCl led to a reduction in liver enzymes, indicating repair of liver damage. H&E and Masson’s trichrome staining revealed a reduction in liver fibrosis post-transplantation, although inflammation persisted. Notably, transplantation of MSC-derived hepatocytes pre-treated with quercetin or combined with rutin and LiCl not only reduced liver fibrosis but also decreased inflammation compared to the untreated MSC group (vehicle). Notably, the quercetin treatment group demonstrated superior liver regeneration and reduced liver fibrosis and inflammation in the acute injury model. However, the therapeutic potential of these cells in chronic liver disease remains to be established. Caution is warranted when extrapolating these findings to chronic liver injury, as the hostile fibrotic microenvironment is known to impair donor cell engraftment, potentially limiting the regenerative capacity of transplanted cells. These findings align with past studies demonstrating that systemic administration of quercetin can alleviate inflammation and fibrosis in mouse livers [34].

Our study uniquely achieved comparable effects by treating MSCs with quercetin ex vivo. We observed that albumin expression, a marker of mature hepatocytes, was highest in quercetin-treated cells. In contrast, combining quercetin with rutin or LiCl increased the expression of alpha-fetoprotein (AFP), an early hepatic marker, suggesting a delay in hepatocyte maturation and limiting the transition to fully mature hepatocytes. These findings suggest that quercetin promotes the generation of more mature and functionally competent hepatocyte-like cells, which may contribute to the superior liver regeneration observed in the quercetin-treated group. Cell tracking revealed that labeled cells were primarily localized in the liver and spleen, with only a few cells found in the kidney and heart, implying that both treated and untreated cells migrated and integrated into the liver.

The use of small molecules to modulate signaling pathways for differentiating stem cells is a promising strategy in regenerative medicine. For instance, IC-2, a small molecule known to inhibit the Wnt/β-catenin signaling in human colorectal cancer cells, induced hepatic differentiation in MSCs [35]. Similarly, salvianolic acid enhanced hepatocyte differentiation of human ESCs through the activation of the Wnt pathway and inhibition of the Notch pathway [36]. Our results suggest that initial downregulation of the Wnt pathway, followed by its upregulation, helped to induce hepatic differentiation. This agrees with the study of Ke et al. [37], who reported that inhibiting the Wnt pathway can promote the differentiation of stem cells into hepatocytes.

Our data showed that transplantation of the pre-treated MSC-derived hepatocytes in the BDL liver fibrosis model reduced liver fibrosis and inflammation better than the MSCs, indicating their potential use in cell-based therapeutics for liver diseases.

## 5. Conclusions

In conclusion, our findings identify ex vivo modulation of MSCs using Wnt pathway regulators, specifically quercetin, rutin, and LiCl, as an effective and previously underexplored strategy to enhance liver regeneration. Among these, quercetin-mediated conditioning was particularly notable, as hepatocytes derived under this condition not only supported robust regenerative responses but also attenuated fibrosis and inflammation. These results suggest that targeted ex vivo priming with natural compounds, either alone or in combination, can direct MSC differentiation towards a hepatocyte lineage with improved therapeutic functionality. Importantly, given the potential systemic adverse effects associated with direct administration of these agents including cardiovascular and gastrointestinal disturbances, this ex vivo approach offers a more controlled and potentially safer alternative. Collectively, our study highlights a promising and translationally relevant strategy for advancing MSC-based therapies in liver disease.

## Figures and Tables

**Table 1:** Primer sequences and their product sizes.

Primer Sequence 5′-3′		Product Size
*Albumin*		
Forward Primer	AGCTGTCCGTCAGAGGATGA	302
Reverse Primer	TCTCAGCGAGACACTGGGAT	
*LDLR*		
Forward Primer	CGGCCACCAGTGTGAAGATA	428
Reverse Primer	CCGTCCAGTAGATGTTGCCA	
*FoxA2*		
Forward Primer	GCTAAGCGGGGCTTCCTG	204
Reverse Primer	GGCTCGTGCCCTTCCATC	
*AFP*		
Forward Primer	GCCTGAAATGACAGAGGAGCA	244
Reverse Primer	CCACATGGAAGTCTCCACCAG	
*CK-18*		
Forward Primer	GACTCCAGCAACTCCTAGCAA	110
Reverse Primer	TGAGCCTTAGTGCCTCAGAAC	
*PPARD*		
Forward Primer	TCAGGGTTAGAAGCTACACAGC	126
Reverse Primer	CCGGGAGAAGAAAGAACAGCTA	
*Wnt*		
Forward Primer	TTGATTTCCAGTGTCCAGAGGG	148
Reverse Primer	ACCTAGTTGGCAATCTCTGCAA	
*β* *-catenin*		
Forward Primer	GTTGAGCTGACCAGTTCCCT	106
Reverse Primer	GGCGATATCCAAGGGCTTCT	
*Axin*		
Forward Primer	CAGATCCGGGAGGATGAAGAAA	108
Reverse Primer	TTCATAGCTGCCAGAGGGTAAG	
*TCF*		
Forward Primer	ACACAAAGCAAACATTATTGGTCA	111
Reverse Primer	CTGAAGATCAGCTACTGGTTACAT	
*GAPDH*		
Forward Primer	GGAAAGCTGTGGCGTGATGG	414
Reverse Primer	GTAGGCCATGAGGTCCACCA	

Low-density lipoprotein receptor (*LDLR*), Forkhead box protein A2 (*FoxA2*), Alpha Fetoprotein (*AFP*), Cytokeratin 18 (*CK-18*), Glyceraldehyde-3-phosphate dehydrogenase (*GAPDH*), Peroxisome proliferator-activated receptor (*PPARD*), T cell factor (*TCF*).

**Table 2:** Treatment of compounds.

Compound Treatment	Time Points (Days)
Quercetin (Q14)	14
Rutin (R14)	14
Lithium chloride (LiCl)	14
Rutin + Quercetin (RQ)	10 + 4
Rutin + Lithium chloride (RL)	10 + 4
Quercetin + Rutin (QR)	10 + 4
Quercetin + Lithium chloride (QL)	10 + 4
Lithium chloride + Quercetin (LQ)	10 + 4
Lithium chloride + Rutin (LR)	10 + 4

## Data Availability

The raw data supporting the conclusions of this article will be made available by the authors on reasonable request.

## Abbreviations

The following abbreviations are used in this manuscript:

AFP	Alpha Fetoprotein
BDL	Bile duct ligation
CK-18	Cytokeratin 18
GGT	Gamma glutamyl transferase
GAPDH	Glyceraldehyde-3-phosphate dehydrogenase
HNF	Hepatocyte nuclear factors
LiCl	Lithium chloride
LDLR	Low-density lipoprotein receptor
MSCs	Mesenchymal stem cells
PPARD	Peroxisome proliferator-activated receptor delta
SGPT	Serum glutamate pyruvate transaminase
TCF	T cell factor
Wnt	Wingless-related integration site
